# The Expanding Role of Quantitative Pupillometry in the Evaluation and Management of Traumatic Brain Injury

**DOI:** 10.3389/fneur.2021.685313

**Published:** 2021-07-12

**Authors:** Jason H. Boulter, Margaret M. Shields, Melissa R. Meister, Gregory Murtha, Brian P. Curry, Bradley A. Dengler

**Affiliations:** ^1^Division of Neurosurgery, Walter Reed National Military Medical Center, Bethesda, MD, United States; ^2^School of Medicine, Uniformed Services University, Bethesda, MD, United States

**Keywords:** quantitative pupillometry, automated infrared pupillometry, pupillary examination, traumatic brain injury, coma, neurocritical care

## Abstract

Traumatic brain injury is a rapidly increasing source of morbidity and mortality across the world. As such, the evaluation and management of traumatic brain injuries ranging from mild to severe are under active investigation. Over the last two decades, quantitative pupillometry has been increasingly found to be useful in both the immediate evaluation and ongoing management of traumatic brain injured patients. Given these findings and the portability and ease of use of modern pupillometers, further adoption and deployment of quantitative pupillometers into the preclinical and hospital settings of both resource rich and medically austere environments.

## Introduction

Traumatic brain injury (TBI) represents one of the world's most prevalent causes of morbidity and mortality. With recent estimates placing its worldwide annual incidence at 64–74 million cases per year, it far exceeds rates of other diseases such as tuberculosis (8.8 million/year) and stroke (16.9 million/year) (1). Of these, resource-poor and medically austere areas are the most affected with low-middle income countries being responsible for three times the number of TBI cases as high income countries (1). Within the Department of Defense (DoD) alone, there have been 413,858 TBIs since 2000 (2).

While TBI management in resource-wealthy areas is driven by imaging, intracranial monitoring, and neurological intensive care units (NICU), management of these injuries in both resource-poor areas and in the non/pre-hospital setting has significant limitations when compared to such an expensive paradigm. Mild TBI (mTBI), for example, is often diagnosed and followed through serial examinations, subjective complaints, and tools such as the standard assessment of concussion (SAC) (3) and military acute concussion evaluation 2 (MACE2) (4). Meanwhile, initial management for moderate/severe TBI (mod/sTBI) is often driven by a TBI severity estimate in the form of the patient's Glasgow coma scale (GCS) score despite known limitations such as a lack of long-term prognostic accuracy (5–7). Furthermore, definitive care may require lengthy and costly medical transportation that not only limits accessibility to care, but also makes the decision of who to transport all the more critical.

Quantitative pupillometry (QP) is rapidly emerging as an effective, affordable, easy-to-use, and reliable tool to diagnose and follow patients with TBIs. The purpose of this Perspective is to advocate for the expanded use of QP in the evaluation and management of patients with TBIs. Deployment of these devices and the accurate use and interpretation of the data they provide offers the potential to improve clinical outcomes in TBI patients at both resource-rich settings such as well-developed intensive care units and resource limited areas such as the preclinical and military forward deployed settings.

## Pupillary Light Reflex Physiology

The pupillary light reflex consists of a complex relationship between photoreceptor activity levels and the pupillary radial and sphincter muscles which is mediated by both cranial nerves II and III. Visible light enters the eye through the iris and strikes the retina where it is converted to an electrical impulse by the rod and cone cells. This information is then refined by bipolar cells and transmitted through the layers of the retina until it leaves the retina as the axons of the ganglion cells which, together, form the optic nerve. The optic nerve proceeds posteriorly through the optic chiasm where the nasal retinal fibers decussate before the information continues posteriorly as the optic tracts. En route to the primary visual cortex, each optic tract gives off a pupillary fiber that travels to the pretectal nucleus of the midbrain. The pretectal nucleus then sends fibers which synapse bilaterally with a preganglionic parasympathetic nucleus called the Edinger-Westphal nucleus. The pretectal nerve then alters the baseline level of activity of the nucleus such that increasing levels of optic tract activity (representing increasing levels of light striking the retina) results in increased activity in the Edinger-Westphal nucleus (8).

The output of the Edinger-Westphal nucleus is what ultimately controls the size of the pupil during the pupillary light reflex. These preganglionic parasympathetic fibers exit the midbrain and travel along bilateral cranial nerve III's to reach the ciliary ganglion. This bilateral output from each Edinger-Westphal nucleus is what is responsible for consensual pupillary constriction after a unilateral light stimulus is applied. After synapsing on the ganglion, postganglionic parasympathetic short ciliary nerve fibers then innervate the iris sphincter muscles and lead to pupillary constriction (8). Of note, further complexity and variability in the pupillary light reflex is introduced by the contraction strength of the antagonistic radial muscle and the impact that a variety of circulating hormones, medications, and/or pathophysiological changes can have on the functionality of the involved neurons and muscles.

## Pupillometry Function

Quantitative pupillometers are small, portable devices which utilize a combination of infrared and visible light to capture this variability in the pupillary light reflexes. At the outset of each measurement, the device measures the pupil at rest with infrared light which produces no pupillary response, and then measures the pupil throughout the entire pupillary light reflex to a flash of visible light. These devices measure pupil baseline size, constriction latency, constriction velocity, constriction percentage, minimum size, and dilation velocity (9). These variables can then be combined into the neurological pupil index (NPi), which is a NeuroOptics proprietary algorithm found in their pupillometers designed to provide a simple numerical understanding of pupillary function (0–5 with scores < 3 considered abnormal) (10).

In addition to data collection, modern pupillometers have also been designed to store the data, produce graphical representations over a period of time, and transmit the data to the electronic medical record making tracking a patient's pupillary responses and NPi's over time easy and useful. Furthermore, the devices themselves are portable and easy-to-use making them well-adapted for distribution to both neurological intensive care units and the forwardly deployed locations of military healthcare and global first responders where these injuries occur.

## Pupillometry Use

### mTBI

Of the predicted 64–74 million TBIs per year, it is estimated that ~81%, or 52–60 million, are mTBIs (1). Populations at some of the highest levels of risk for mTBIs include youth competitive athletes and military personnel. Unfortunately, mTBI screening among these groups often relies at least in part on self-reported symptoms. This introduces a lack of sensitivity due to inherent survey issues, inter-rater variability, and even a lack of patient honesty with regard to symptoms (11, 12). In youth sports alone, it is estimated there are ~500,000–1.2 million untreated concussions annually (13). The lack of detection of these concussions becomes increasingly more important as both pre-clinical and clinical data continue to aggregate demonstrating that repeated concussions are associated with neuroinflammation, cortical loss, and behavioral changes (14–16).

Here, QP represents a potential mechanism for both diagnosing a mTBI and following patients as they recover. Research in adolescent athletes has demonstrated changes in pupillometry following concussive events. One such study utilizing football helmet accelerometers to accurately define mTBIs found that immediately following mTBIs, QP demonstrated alterations in the pupillary light reflex from baseline testing. Specifically, the affected athletes were noted to have decreased pupillary constriction, a reduction in both constriction and dilation velocity, and a trend toward a decreased NPi (with the lack of significance likely limited by the sample size of 13). Comparatively, QP of the same athletes immediately following a game without a mTBI demonstrated only a statistically significant *increase* in constriction velocity (17). Taken together, these results demonstrate both a rapid, objective manner in which to diagnose acute mTBIs that appears to not be confounded by the altered sympathetic tone patients may be experiencing at the time.

These alterations in the pupillary light reflex have also been demonstrated in the subacute mTBI period. High school athletes recruited from high school concussion programs within 1 month of injury demonstrated a larger pupillary maximum and minimum diameter and a greater percent constriction when compared to controls (18). Within military personnel who were 2–6 weeks from a diagnosed mTBI, QP demonstrated longer constriction latency and a slower constriction and dilation velocity when compared to patients without a mTBI (19).

While each of these studies demonstrate a different group of changes following a mTBI, the presence of these alterations clearly allows for mTBI detection and monitoring through recovery. Further research will be needed to ensure not only that there is an accurate standard from which to compare, but also to better delineate which aspects of the pupillary light reflex and which timepoints during recovery from a mTBI should be evaluated. Nevertheless, the present data suggests that mTBI detection and monitoring would benefit from increased utilization of QP.

### Mod/sTBI

While significantly less ubiquitous than mTBI, mod/sTBI represents no less of a medical challenge. With a median hospitalization cost of $55,267 per case and an estimated 5.5 million cases of mod/sTBI annually, ensuring optimal outcomes for invested resources represents a high value decision point (1, 20). Within the military, this decision is even more important as forward-deployed settings are not research rich and accurate medical triage can become crucial in maximizing medical benefit through ensuring limited waste during medically taxing mass casualty events. Research within the last two decades has demonstrated that QP offers benefit to many aspects of the care for a mod/sTBI patient.

The classical neurological trauma examination includes two parts: the GCS score and the pupillary examination. While a patient's GCS score can be replicated with reasonable consistency, multiple reports have demonstrated that the same cannot be said for manual evaluation of the neurologically injured patient's pupillary reactivity. For example, a recent study demonstrated a 22% error rate amongst nurses in detecting anisocoria when compared to QP (9). Another study comparing nursing staff in a neurological intensive care unit to QP, found that the nursing staff had a 50% error rate in correctly diagnosing anisocoria and a 20% error rate in determining pupillary reactivity. In patients with pupils <2 mm in diameter, as may be the case following drug intoxication or medication administration for intubation and stabilization, the error rate increased to 39% (21). Despite this increase in nursing/provider error rate, multiple studies have demonstrated that QP is still able to detect the presence of pupillary constriction in spite of multiple medications including paralytics, opioids, and benzodiazepines (22). In these situations, QP removes this potential for error and instead provides a rapid and reliable physical examination finding.

Such speed and accuracy becomes increasingly important when discussing surgical decision making for the mod/sTBI patient, especially for those worse off. While the GCS's ease of use contributes to its wide use in trauma evaluations, limitations regarding its ability to prognosticate, and therefore drive treatment decisions, have been raised (23, 24). An increasing number of studies have demonstrated the prognostic importance of pupillary reactivity in delineating patients with a GCS score of three (5–7). In these studies specifically, stratification of patients with a GCS of three by pupillary light examination resulted in an identifiable difference in outcome. Patients who had at least one reactive pupil had a mortality rate of 40–50% compared to the 100% mortality rate of those who had bilaterally fixed and dilated pupils (5, 7). Indeed, the addition of the pupillary examination to the GCS score has been found to be significantly more predictive of mortality than GCS alone (6). This utility for QP, as an intervention adjudicator, was recently evaluated in a pilot study of neurotrauma patients. The study demonstrated that all patients who underwent monitor placement in accordance with the 4th edition Brain Trauma Foundation guidelines but had an NPi of >3 had the monitor removed in <24 h (25). Further study will be required, but the ability to easily obtain such highly accurate pupillary information could help identify and expedite those who can benefit from neurosurgical intervention.

QP has also demonstrated utility in the management of mod/sTBI patients within the critical care unit. The management of the mod/sTBI patient is often dictated by frequent neurological evaluations and intermittent head CTs in response to neurological changes. QP has been demonstrated to detect episodes of increased intracranial pressure, specifically uncal herniation, prior to the onset of clinical symptoms (26, 27). While not perfect due to findings such as the Kernohan's notch phenomenon, the abnormalities detected by QP are also predictive of the sidedness of the lesion (26, 28). In addition to accelerating detection of neurological decompensation, QP has also been utilized to help guide the use of hypertonic therapy. Two studies recently demonstrated that abnormal NPi's in patients with increased intracranial pressure could be reduced or normalized with hypertonic therapy thereby providing a non-invasive way to monitor effect of hypertonics (29, 30).

## Conclusion

TBI represents a rapidly increasing pathology on a global scale. As both mTBIs and mod/sTBIs continue to increase in prevalence and we understand more regarding the implications of these injuries, identification and management in both the immediate and post-injury setting will become more important. Over the last two decades numerous studies have demonstrated the potential for QP to assist in the detection and management of TBI across its spectrum of severity and the longitude of its course. This utility coupled with the ease of QP and the durability of today's pupillometers make QP a resource that is easy to expand across countries of varying income levels and practice areas of varying levels of austerity.

## Data Availability Statement

The original contributions presented in the study are included in the article/supplementary material, further inquiries can be directed to the corresponding author/s.

## Author Contributions

JB, MS, and BD developed the idea for and organized the manuscript. JB, MS, MM, GM, BC, and BD wrote, revised, and critically evaluated the manuscript. All authors contributed to the article and approved the submitted version.

## Conflict of Interest

The authors declare that the research was conducted in the absence of any commercial or financial relationships that could be construed as a potential conflict of interest.
